# Frailty as a Moderator of the Relationship Between Social Isolation and Health Outcomes

**DOI:** 10.1093/geroni/igaa057.863

**Published:** 2020-12-16

**Authors:** Fereshteh Mehrabi, François Béland

**Affiliations:** University of Montreal, Montreal, Quebec, Canada

## Abstract

Previous studies have reported that social isolation is a predictor of adverse outcomes, which is also closely associated with frailty. Very little is known about the moderating role of frailty on the impact of social isolation on health. We performed a cross-sectional analysis of the first wave of the FRéLE longitudinal study, consisting of 1643 Canadian community-dwelling older adults aged 65 years and over. Multivariate regression analysis was performed to examine the interaction between social isolation and frailty on health, controlling for socioeconomic characteristics and life habits. Social isolation was measured through social participation, social networks and support for different social ties namely, friends, children, extended family, and partner. In contrast to Berkman’s theory on the impact of social isolation on health, we found that frailty had no modifying role on the effects of social isolation on health. Frailty was significantly associated with all adverse outcomes. Less social participation was associated with ADLs, IADLs, depression and cognitive decline. The absence of friends was associated with depression and cognitive decline. Less support from children and having no children were associated with ADLs, comorbidity and depression. Fewer contact with extended family and having no family members were notably associated with ADLs and IADLs. Those who received less support from a partner or had no partner were more depressed and had more difficulties in performing IADLs. This study suggests that older adults who participate in social activities and have social ties, feel better with respect to physical health than those who feel isolated.

